# Voxelwise statistical methods to localize practice variation in brain tumor surgery

**DOI:** 10.1371/journal.pone.0222939

**Published:** 2019-09-27

**Authors:** Roelant Eijgelaar, Philip C. De Witt Hamer, Carel F. W. Peeters, Frederik Barkhof, Marcel van Herk, Marnix G. Witte

**Affiliations:** 1 Cluster Radiation Oncology, The Netherlands Cancer Institute, Amsterdam, The Netherlands; 2 Neurosurgical Center Amsterdam, Brain Tumor Center Amsterdam, Amsterdam University Medical Center, location VUmc, Amsterdam, The Netherlands; 3 Department of Epidemiology & Biostatistics, Amsterdam Public Health Research Institute, Amsterdam University Medical Centers, location VUmc, Amsterdam, The Netherlands; 4 Department of Radiology & Nuclear Medicine, Amsterdam University Medical Center, Location VUmc, Amsterdam, The Netherlands; 5 Institutes of Neurology & Healthcare Engineering, University College London, London, United Kingdom; 6 Division of Cancer Sciences, Manchester Cancer Research Centre, School of Medical Sciences, Faculty of Biology, Medicine & Health, University of Manchester, Manchester Academic Health Sciences Centre, Manchester, United Kingdom; University of North Carolina at Chapel Hill, UNITED STATES

## Abstract

**Purpose:**

During resections of brain tumors, neurosurgeons have to weigh the risk between residual tumor and damage to brain functions. Different perspectives on these risks result in practice variation. We present statistical methods to localize differences in extent of resection between institutions which should enable to reveal brain regions affected by such practice variation.

**Methods:**

Synthetic data were generated by simulating spheres for brain, tumors, resection cavities, and an effect region in which a likelihood of surgical avoidance could be varied between institutions. Three statistical methods were investigated: a non-parametric permutation based approach, Fisher’s exact test, and a full Bayesian Markov chain Monte Carlo (MCMC) model. For all three methods the false discovery rate (FDR) was determined as a function of the cut-off value for the *q*-value or the highest density interval, and receiver operating characteristic and precision recall curves were created. Sensitivity to variations in the parameters of the synthetic model were investigated. Finally, all these methods were applied to retrospectively collected data of 77 brain tumor resections in two academic hospitals.

**Results:**

Fisher’s method provided an accurate estimation of observed FDR in the synthetic data, whereas the permutation approach was too liberal and underestimated FDR. AUC values were similar for Fisher and Bayes methods, and superior to the permutation approach. Fisher’s method deteriorated and became too liberal for reduced tumor size, a smaller size of the effect region, a lower overall extent of resection, fewer patients per cohort, and a smaller discrepancy in surgical avoidance probabilities between the different surgical practices. In the retrospective patient data, all three methods identified a similar effect region, with lower estimated FDR in Fisher’s method than using the permutation method.

**Conclusions:**

Differences in surgical practice may be detected using voxel statistics. Fisher’s test provides a fast method to localize differences but could underestimate true FDR. Bayesian MCMC is more flexible and easily extendable, and leads to similar results, but at increased computational cost.

## Introduction

Despite intensive chemotherapy, radiotherapy, and surgery regimens, prognosis for glioblastoma patients remains poor. Further escalation of treatment might improve disease specific survival, but would likely come at the expense of increased toxicities, reducing quality of life. For a surgeon performing a glioblastoma resection the dilemma is where to stop tumor removal. Stopping early may incur earlier tumor recurrence, while stopping late may compromise tumor-infiltrated functional brain tissues. Such decisions are generally discussed within a surgical team, based on the available MR imaging and clinical status. The result of such a discussion is a surgical plan, aiming for the maximum extent of resection attainable for that patient. However, another team presented with the same patient might well make other decisions, and come to a different surgical plan. For a single patient it is not possible to say which plan would be superior, but using larger cohorts of patients it should be possible to compare the surgical practices of two teams and learn how to strike a better compromise between tumor control and toxicity.

The difficulty in such comparisons is that tumors have different shapes and sizes for each patient. To address this, we proposed the use of resection probability maps [1], in which surgical results (tumor and residue) are aggregated in atlas space over cohorts of patients. In such a resection probability map it is essentially assumed that the likelihood of a piece of tumor to be removed depends on its anatomical location only. Identified differences in the probability of tumor removal at a given anatomical location can help focus further research to determine if either pattern is associated with improved outcome in multivariate analyses. This data could consequently help to arrive at consensus of the best practice for patients with a tumor in that region. Ultimately, the best practice should be based on a collaborative body of objective data, rather than the intuition of a surgical team.

In a previous publication [2], a proof of concept was presented for the use of resection probability maps to compare and discuss the patterns of current care. In that work, the metric for comparison was a non-parametric permutation approach based on ‘risk-minus’ values, defined as the voxelwise difference in resection probabilities between practices. Differentially resected regions between two hospitals were identified in the anterior limb of the right internal capsule and the right caudate nucleus, indicating practice variation. The permutation method in that work was designed to provide a *q*-value that could be thresholded into a set of voxels with a given estimated false discovery rate [3, 4], however its validity had not yet been investigated. In brain tumor resection, the presence of residual tumor after surgery in a voxel is subject to the presence of tumor before surgery, and the tumor location varies from patient to patient. This means that discrete statistics should be applied, and that the number of experiment repetitions varies from voxel to voxel. This complicates the correction for multiple testing, and sets the voxel statistics of brain tumor resections apart from those of e.g. voxel-based lesion-symptom mapping [5, 6]. Resection probability maps provide information about the ratios of residues and tumors rather than their absolute numbers, and so does the permutation statistic based on risk-minus. In an attempt to limit the bias which might be introduced by this underlying discreteness, the permutation statistic only considered voxels with sufficiently large numbers of observed tumors (6 or more).

In another study the Fisher’s exact test was used as metric for comparison of resection probability maps, combined with a permutation based multiple testing correction. [1] This metric does take into account the absolute numbers of residues and tumors, however its validity had not yet been investigated either.

In this work we generated synthetic data from a model to determine the accuracy of the permutation statistic and compared this to the Fisher’s exact test [1] and a full Bayesian Markov Chain Monte Carlo approach. The proposed full Bayesian approach uses a hierarchical model and tests the probability of zero effect falling inside the largest highest density interval (HDI) of the difference between the estimated success rates. Benchmarking these methods will aid to evaluate variations in localized extent of resection in clinical practice using large patient registries.

## Methods and materials

Custom software modules were written in C++ and connected using a Python scripting layer.

### False discovery rate using a permutation approach

For two patient cohorts from practices A and B, the numbers of tumors *t* and residues *r* for each brain voxel were stored as a vector of rational numbers. Elements with fewer than 6 patients (*t*_A_ + *t*_B_ < 6) were removed. Risk-minus values for the remaining elements were evaluated as $\left|\frac{r_{A}}{t_{A}}-\frac{r_{B}}{t_{B}}\right|$. Next, permutations were performed by shuffling (relabeling) patients between practices. Risk-minus values over all elements and all (1000) permutations were collected in a cumulative histogram, representing the null distribution. *p*-values per element were derived by looking up the cumulative count at the observed risk-minus. A computationally efficient implementation of this histogram was created using rational number arithmetic and a C++ map structure. A *q*-value estimating the FDR was evaluated. For each unique *p*-value the number of falsely declared significant voxels was estimated from the cumulative histogram of *p*-values and divided by the number of declared voxels at that *p*.

### False discovery rate using Fisher’s exact test

A second method was implemented to compute the exact null distribution over all voxels, thereby explicitly taking into account that measurements are discrete (and typically small), and that the numbers of measurements differ between voxels. For each voxel the numbers of observed tumors and residues per practice can be summarized in Table 1. For fixed marginals (i.e. numbers of tumors per practice *t*_A_, *t*_B_ and total number of residues *r* per voxel), the number of residues in practice A (*r*_A_) follows the hypergeometric distribution under the null hypothesis (no difference in resection probability between the practices), and *p*-values can be derived using Fisher’s exact test. The two-sided *p* for an observed *r*_A,obs_ was computed by first evaluating the hypergeometric probability Pr(*r*_A_) for each possible observation 0 ≤ *r*_A_ ≤ *r*, and a one-sided *p* evaluated as the smaller of $\sum_{0}^{r_{A,obs}}\text{Pr}(r_{A})$ and $\sum_{r_{A,obs}}^{r}\text{Pr}(r_{A})$. To form a two-sided *p*, values of Pr(*r*_A_) smaller than the one for the observed *r*_A_ value were added from the opposing side (starting at either 0 or *r*), terminating just before the resulting *p* would exceed twice the value of the single sided *p* [7]. Next, a cumulative null histogram of all possible *p*(*r*_A_) values over all tested voxels was constructed to estimate the expected abundance of *p*-values under the null hypothesis [8, 9]. *q*-values were determined from these as $q=\frac{N_{0}f_{0}}{i}=\frac{(N-N_{a})f_{0}}{i}$, with *N*_0_ the true number of null voxels, *N*_*a*_ the number of true associated voxels, *N* the total number of voxels, *f*_0_ the fraction falsely declared non-null (at the given cut-off value *q*), and *i* the number of voxels declared associated. Noting that *N*_*a*_ = *i* − *iq*, we can solve $q=\frac{f_{0}}{1-f_{0}}\frac{N-i}{i}$.

**Table 1:** Contingency table for the observed numbers of tumors *t* and residues *r* in a voxel.

Residue	Practice		Total
	A	B	
Yes	*r*_A_	*r*_B_	*r*
No	*t*_A_ − *r*_A_	*t*_B_ − *r*_B_	*t* − *r*
Total	*t*_A_	*t*_B_	*t*

### Bayesian model with partial pooling for repeated binary trials

We set up a full Bayesian model for Markov chain Monte Carlo sampling using a no-U-turn sampler in the Stan software package [10]. A hierarchical model was used which treats voxels as belonging to a population, estimating the properties of this population along with voxel properties. The amount of pooling between voxels is thus implicitly controlled, which is referred to as partial pooling (see http://mc-stan.org/users/documentation/case-studies/pool-binary-trials.html). Given the number of patients with a tumor in each institution, *t*_A_ and *t*_B_, and the number of observed residues, *r*_A_ and *r*_B_, a trial for each institution is observed in a voxel with a success rate of $\frac{t_{A}-r_{A}}{t_{A}}$ and $\frac{t_{B}-r_{B}}{t_{B}}$. The parameter of interest was the difference between these success rates of institutions in voxels. Given the success rates and the number of tumors, the number of residues was represented by a binomial distribution. The logit-transformed overall success rate *μ* was assumed to be normally distributed with unknown mean and standard deviation, using mean 0 and standard deviation 1 as vague priors. For efficiency of convergence, a non-centered parameterization was adopted for voxelwise random effects with mean 0 and standard deviation 1 [11]. Vague priors were chosen to primarily reflect inference from the observed data without substantive prior knowledge. The Stan code that was used is listed in the supporting information. Four parallel chains of 500 Hamiltonian Monte Carlo simulation samples were executed, of which the first 50% were discarded as burn-in. The remaining 50% (1000 samples in total) were considered to be draws from the posterior distribution. An estimator for the difference in success rates in a voxel was computed by evaluating the probability level of the largest highest density interval (HDI) centered within the posterior difference distribution which would exclude zero difference. The potential multiple comparison problem is addressed by the partial pooling because the estimates for differences are shrunk towards the background institutional success rate [12].

### Synthetic tumor resection data

**Fig 1:**
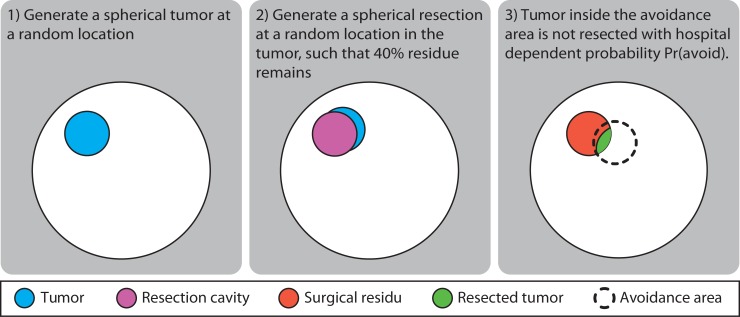
Schematic illustration of the generative model for brain tumor resections.

Fig 1 shows a schematic illustration of the model used to generate generate synthetic data on which to test the statistical methods. A spherical brain was created with the same number of voxels (1.7M) as the brain mask of a 1 mm resolution MNI atlas [13]. To simulate surgical treatment of a patient, a spherical tumor was generated with its center at a random location within this spherical brain. Next, a spherical resection cavity was placed with its center at a random location within this tumor, and with a diameter (depending on this location) such that it encompassed a certain fraction of the tumor, thus leaving a crescent residue. Tumor and residue protruding outside the brain sphere were cropped. A spherical surgical avoidance region was designated in which a probability could be set that a planned resection would not be executed. In such a case this avoidance region was removed from the sphere representing the resection cavity before being applied to the tumor. Two patient cohorts were then generated with a different surgical avoidance probability, simulating a different surgical practice. The resulting voxelwise tumor incidence Pr(*t*) and resection probability Pr(*r*) were the same for the two centers, except in the avoidance region where the resection probabilities were Pr(*r*_A_) = Pr(*r*)*Pr(avoid_A_) and Pr(*r*_B_) = Pr(*r*)*Pr(avoid_B_) in centers A and B, respectively. The model parameters were set to values similar to those observed in clinical practice when including biopsy patients: the number of patients per cohort (50), the tumor diameter (8cm), and the extent of resection (60%). The size of the surgical avoidance region was unknown and set to a sphere of 4 cm diameter. The different voxelwise statistical tests were then performed, and cumulative histograms generated for both the voxels inside and the voxels outside the avoidance region. As a function of the cut-off value for the *q*-value or the highest density interval, the observed false discovery ratio was determined, and receiver operator characteristic (ROC) as well as precision recall (PRC) curves were created; the latter are deemed more important given skewness of the data. By repeating the synthetic model generation 1000 times, confidence intervals on these curves were constructed.

### Patients

The patient cohorts were previously described in [2]. In total 77 adults with first-time surgical (sub)total resection of a right sided glioblastoma in 2012-2013 were selected, resulting in 48 patients treated by neuro-oncological hospital A, and 29 by hospital B. Preoperative tumors and postoperative residues had been segmented on MRI in 3D, registered to standard brain space with a resolution of 1 × 1 × 1 mm, and sub-sampled for analyses to 2 × 2 × 2 mm voxels. Mean tumor volumes were 41 cm^3^ and 48 cm^3^, extents of resection 95% and 96% in hospitals A and B respectively. As these cohorts did not include patients for whom only a biopsy was performed, tumor volumes were smaller and extents of resection larger than on average for the entire population of GBM patients.

## Results

**Fig 2:**
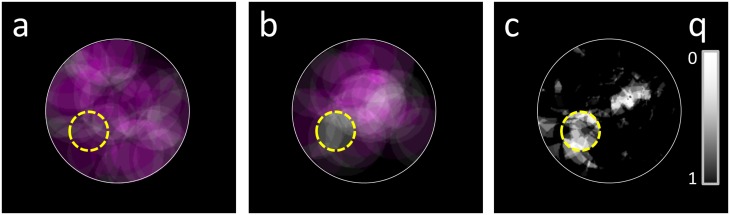
A synthetic model for brain tumor resections. Cumulative maps of tumors (purple) and residues (green) are overlaid for 50 patients in practice A (*a*) and 50 in practice B (*b*). A yellow dashed line marks a spherical region in which practice B does not resect. Locations where most tumors were resected appear purple, locations where all tumor was left as residue show up gray. *c)* Estimated *q*-values using Fisher’s exact test.

Fig 2 shows the cumulative tumor and residue maps for the case of 0% avoidance (i.e. no difference with surrounding brain) in practice A, and 100% avoidance (i.e. tumor is always left as residue) in practice B. As an example, the resulting *q*-map based on Fisher’s exact test for these data is shown in panel Fig 2c, which can be used to evaluate the numbers of true and false positives as a function of *q*-cut-off. Voxels inside the boundaries of the effect region mainly have low *q*-values and contribute to true positives, however some appear dark and lead to false negatives. On the other hand, outside the effect region some clusters of voxels acquired low *q*-values by chance, resulting in false positive detections.

The computational effort for the three statistical methods varied considerably, and at the full scan resolution of 1 mm^3^ voxels proved prohibitive for both the permutation and Bayesian methods. Therefore, a sub-sampling to voxels of 4 × 4 × 4 mm was performed. For 1000 repetitions of the synthetic data generating model this resulted in computation times on a dual ten-core Intel Xeon workstation on the order of minutes for Fisher’s method, hours for the permutation method, and weeks for the Bayesian method. Consequently, further evaluations of variations of the generative model parameters were only feasible with Fisher’s method. In S1 Fig in the supporting information results of Fisher’s test for 2 × 2 × 2 mm and 1 × 1 × 1 mm voxel resolutions are demonstrated.

### Permutation approach

**Fig 3:**
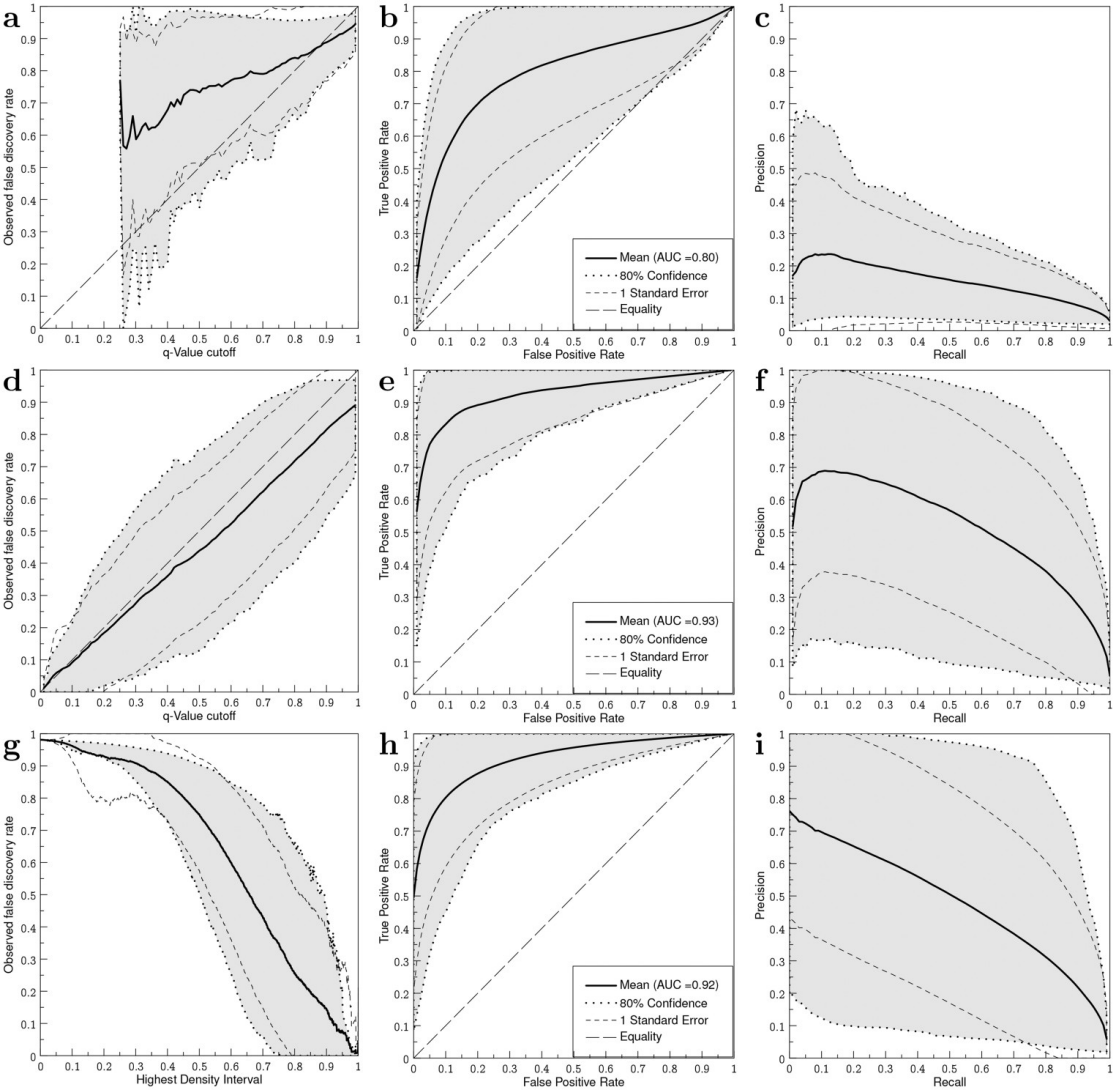
Statistics for synthetic tumor resection data. *a)* Observed FDR vs. cut-off on the *q*-value using a permutation approach. *b)* and *c)* Corresponding ROC and PRC curves.*d)* Observed FDR vs. cut-off on the *q*-value using Fisher’s test. *e)* and *f)* Corresponding ROC and PRC curves. *g)* Observed FDR vs. cut-off on the highest density interval excluding 0 for a hierarchical full Bayesian model. *h)* and *i)* Corresponding ROC and PRC curves.

Observed FDR for the permutation approach indicated that the estimated *q*-values were too liberal (Fig 3a). The method rarely generated *q*-values below 0.35 and never below the value of 0.25, which resulted in the jagged appearance of the curve. The ROC curve (Fig 3b) still shows a predictive power for the model, with an AUC of 0.80, however the PRC curve (Fig 3c) indicates it has quite low precision.

### Fisher’s test

Corresponding results for Fisher’s test show that the estimated *q*-values on average provide a slightly conservative estimate of observed FDR (Fig 3d). ROC and PRC curves (Fig 3e and 3f) show an improved performance compared to the permutation approach, with an AUC of 0.93.

### Bayesian Monte Carlo

As a small highest density interval which excludes zero difference implies a difference distribution which is centered closely to zero, setting a low HDI probability level threshold results in high FDR. Fig 3g therefore shows a declining curve. Performance was similar to Fisher’s method in terms of ROC (Fig 3h) and PRC Fig 3i), with a similarly high AUC of 0.92.

### Synthetic model parameter variations

To illustrate the applicability of the statistical tests to data sets with other characteristics, Fisher’s test was repeated for several variations of the synthetic model. S2 Fig through S7 Fig in the supporting information show the FDR, ROC and PRC curves for these results. Better accuracies were observed for larger tumors (S2b Fig), an increased patient cohort size (S3b Fig), larger effect region size in which a difference in practice exists (S4b Fig), and increased extent of resection (S5b Fig). On the other hand, overly liberal FDR estimates were seen for *∅* 6 cm tumors (AUC = 0.74, S2a Fig), for cohorts of 25 patients (AUC = 0.78, S3a Fig), when decreasing the region with a difference in practice to 2 cm diameter (AUC = 0.84 S4a Fig), or when using a 30% extent of resection (AUC = 0.69, S5a Fig).

A reduced contrast between the practices’ surgical avoidance probabilities is expected to reduce the ability of the statistical methods to detect the effect region. For both Fisher’s test and the Bayesian method, simulations were performed for surgical avoidance probabilities of 10% versus 90%, 20% versus 80%, 30% versus 70%, and 40% versus 60%. The results are shown in S6 and S7 Figs. Both methods show reduced performance at lower avoidance probability contrast, with somewhat higher AUC values for the Bayesian method.

### Patient data

**Fig 4:**
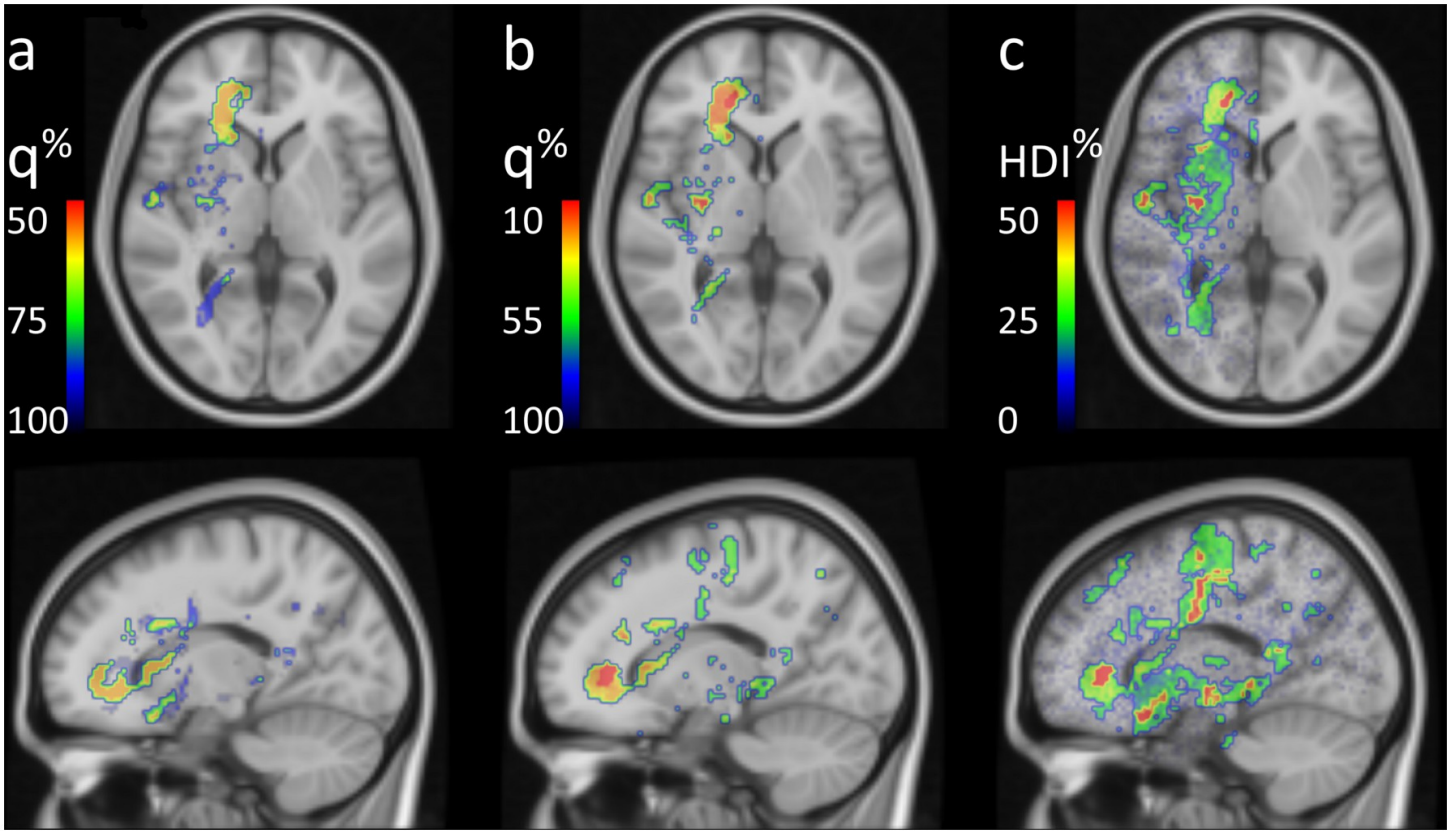
Voxelwise statistical methods detecting differences in surgical practice, showing regions considered significantly different for a given cut-off. *a)* A non-parametric permutation approach and *b)* Fisher’s exact test using a cut-off for estimated FDR, *c)* a full Bayesian model using Markov chain Monte Carlo methods and a cut-off on the probability level of the highest density interval excluding zero.

Application of the three statistical methods to the cohorts of right sided GBM patients is illustrated in Fig 4. For each method cut-off levels were scaled to accommodate the observed values. Notably, the *q* levels found using Fisher’s test were much lower than using the permutations. However, Fisher’s method may underestimate true FDR, depending on the details of the underlying effect. A region with low *q* in Fisher’s test (red in Fig 4b) was left out in the permutation test (Fig 4a) due to the small number of total tumors (<6) in these voxels. In the Bayesian approach this same region also has high HDI, however there are additional regions deeper in the brain which were not prominent in the first two methods yet reach similar HDI probability level (Fig 4c).

## Discussion

In this work we developed an efficient implementation of voxelwise statistical tests for application to the field of radiological evaluation of glioma surgery, thereby enabling an extensive benchmarking of their performances. Application to patient data led to identification of similar regions, however the quantitative assessments of these regions differed between the methods.

The first permutation-based method had been used in previous analyses and was based on permutations of patients between practices. This method was set up to generate a *q* map that could provide estimated false discovery rates. Our results indicate that these were likely underestimated. A methodological drawback of the comparison of risk-minus values is that the number of tests per practice in a voxel is not taken into account in the statistic (one residue out of two tumors has similar weight to ten out of twenty). Furthermore, when performing permutations on a patient level the numbers of tumors per practice are necessarily also shuffled. This implies that the derived statistics will also harbor information about the likelihood of observing a certain asymmetry in the numbers of tumors per practice in a voxel.

These issues were mitigated by considering a method based on Fisher’s exact test, which takes the numbers of tests and observations explicitly into account, and which considers the marginals (total tumor and residue counts) in a voxel to be fixed. FDR was estimated by explicitly calculating expected rates of *p*-values under the null hypothesis. [14] consider the dependency of *p*-values on the marginals and arrive at a similar expression by using a maximum likelihood estimate for the marginals. [15] show that in symptom lesion mapping Fisher’s exact test is overly conservative, whereas the Liebermeister test (considering non-fixed marginals) provides additional statistical power. In our application to brain surgery we did not observe a conservative tendency of Fisher’s test, possibly due to the way we perform a multiple testing correction by explicitly generating a null distribution as histogram of all possible voxel wise *p* values while constructing the map of *q* values. On the other hand, cases in which the *q* values underestimated observed false discovery rates were seen for less favorable situations, notably for cases with less contrast in surgical avoidance probabilities between practices As this contrast is not known in clinical practice, the quantitative interpretation of a *q* map based on this test should be done with caution. Note also that even though in favorable cases the method provides a near-nominal prediction of FDR on average, there is a wide confidence interval around this prediction, and in about half the cases the prediction would in fact be an underestimation. A major advantage of the Fisher’s method is its calculation speed. The method however is limited to the special case of comparing exactly two cohorts, e.g. surgical practices here. Furthermore, the application of a hypergeometric distribution to each individual voxel does not exploit information about the ensemble of voxels as a whole, which might result in higher power.

The Bayesian model with partial pooling was explored as a more flexible, potentially more powerful alternative. Different from the previous methods which aim to assess the null hypothesis (of no difference in resection probability between practices), the Bayesian model tries to estimate the resection probability per practice in each voxel. The partial pooling causes these probabilities to be similar among voxels, unless the observation in a voxel is systematically different from the others. While pooling has an equalizing effect on the posterior resection probability distributions and replaces the multiple testing correction that was required in the other methods, the exact relation between *q* values and the Bayesian HDI statistic is not easily established. While constructing the Bayesian model it was noticed that exclusion of voxels in which no relevant difference between the practices could be observed (e.g. voxels with tumor but no residue) led to different posterior resection probability distributions, and different HDI intervals. Inclusion of these voxels led to better models, while in Fisher’s test these voxels had no effect on the outcome. This difference in the way non-informative voxels were handled may also have caused the different appearance of the PRC curve towards low recall values between Fisher’s method (Fig 3f), showing a local maximum around a recall value of 0.1, and the Bayesian method (Fig 3i), which shows no local maximum.

As the data consisted of a subset of patients for whom a tumor resection was performed (as opposed to a tumor biopsy), the parameters differed from those considered representative for the entire population of surgical patients. Tumor volumes varied between patients and were smaller than nominal, while the extent of resection was higher. Our analyses indicated that some of these effects may improve, while others may deteriorate the statistical analyses. As a result, there is considerable uncertainty in the quantitative interpretation of the statistical methods applied to clinical data. Also, it should be noted that uncertainties are associated with the delineation of pre- and post-operative tumor [16], and with the deformable registration towards a common atlas space. While uncertainties such as these could be incorporated into the synthetic model to determine their effect on the voxelwise statistics, those investigations are beyond the scope of the current work.

All statistical methods we presented in this work were voxelwise, i.e. correlations between pixels were not incorporated into the statistics. Alternative formulations might leverage the fact that the tumor voxels are spatially clustered [17] and arrive at more powerful statistics, and such methods are worthwhile to be considered in future research.

Based on our results, we propose avoiding the permutation method in future analyses of surgical results for tumor resection. While previous conclusions using this method should still hold, Fisher’s method provides a faster alternative with similar conclusions for comparison between two cohorts. A quantitative interpretation of its *q* values should only be cautiously made. To analyze more complex situations with multiple patient cohorts or voxels within anatomical regions, the flexibility of the Bayesian model may be a suitable alternative, although at the cost of increased computational time.

## Supporting information

S1 Appendix Stan code. (EPS)

S1 Fig Synthetic tumor model variations. Fisher’s test using *a)* 2 × 2 × 2 mm and *b)* 1 × 1 × 1 mm voxels. Other parameters were at nominal values: *∅* 8 cm tumors, patient cohorts of 50 patients, a 60% extent of resection, and a *∅* 4 cm effect region with an avoidance probability difference of 0% versus 100%.
(TIF)

S2 Fig Synthetic tumor model variations. Fisher’s test using *a)* *∅* 6 cm and *b)* *∅* 10 cm tumors. Other parameters were at nominal values: 4 × 4 × 4 mm voxels, patient cohorts of 50 patients, a 60% extent of resection, and a *∅* 4 cm effect region with an avoidance probability difference of 0% versus 100%.
(TIF)

S3 Fig Synthetic tumor model variations. Fisher’s test using *a)* patient cohorts of 25 patients and *b)* patient cohorts of 100 patients. Other parameters were at nominal values: 4 × 4 × 4 mm voxels, *∅* 8 cm tumors, a 60% extent of resection, and a *∅* 4 cm effect region with an avoidance probability difference of 0% versus 100%.
(TIF)

S4 Fig Synthetic tumor model variations. Fisher’s test using *a)* a *∅* 2 cm and *b)* a *∅* 6 cm effect region. Other parameters were at nominal values: 4 × 4 × 4 mm voxels, *∅* 8 cm tumors, patient cohorts of 50 patients, a 60% extent of resection, an avoidance probability difference of 0% versus 100% inside the effect region.
(TIF)

S5 Fig Synthetic tumor model variations. Fisher’s test using *a)* a 60% and *b)* a 90% extent of resection. Other parameters were at nominal values: 4 × 4 × 4 mm voxels, *∅* 8 cm tumors, patient cohorts of 50 patients, and a *∅* 4 cm effect region with an avoidance probability difference of 0% versus 100%.
(TIF)

S6 Fig Synthetic tumor model variations. Fisher’s test using avoidance probability differences of *a)* 10% versus 90%, *b)* 20% versus 80%, *c)* 30% versus 70%, and *d)* 40% versus 60% inside the effect region. Other parameters were at nominal values: 4 × 4 × 4 mm voxels, *∅* 8 cm tumors, patient cohorts of 50 patients, a 60% extent of resection, and a *∅* 4 cm effect region.
(TIF)

S7 Fig Synthetic tumor model variations. The Bayesian method using avoidance probability differences of *a)* 10% versus 90%, *b)* 20% versus 80%, *c)* 30% versus 70%, and *d)* 40% versus 60% inside the effect region. Other parameters were at nominal values: 4 × 4 × 4 mm voxels, *∅* 8 cm tumors, patient cohorts of 50 patients, a 60% extent of resection, and a *∅* 4 cm effect region.
(TIF)
