# Supplementary figures and images for: Voxelwise statistical methods to localize practice variation in brain tumor surgery
Source: PLoS One. 2019 Sep 27;14(9):e0222939. doi: 10.1371/journal.pone.0222939 (PMC6764660; doi:10.1371/journal.pone.0222939)

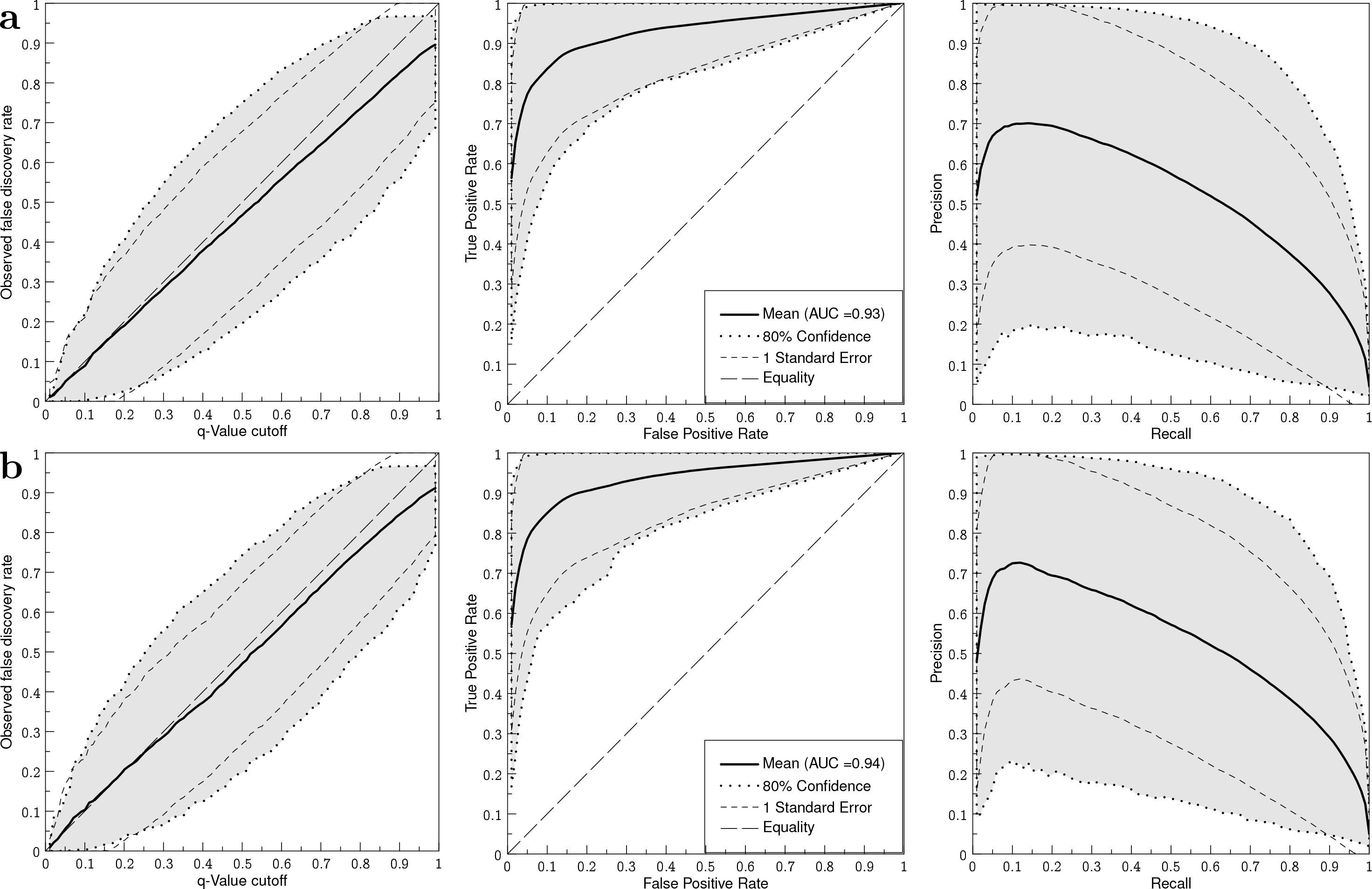

Supplement: S1 Fig — Fisher’s test using a) 2 × 2 × 2 mm and b) 1 × 1 × 1 mm voxels. Other parameters were at nominal values: ∅ 8 cm tumors, patient cohorts of 50 patients, a 60% extent of resection, and a ∅ 4 cm effect region with an avoidance probability difference of 0% versus 100%. (TIF) [file pone.0222939.s002.tif]

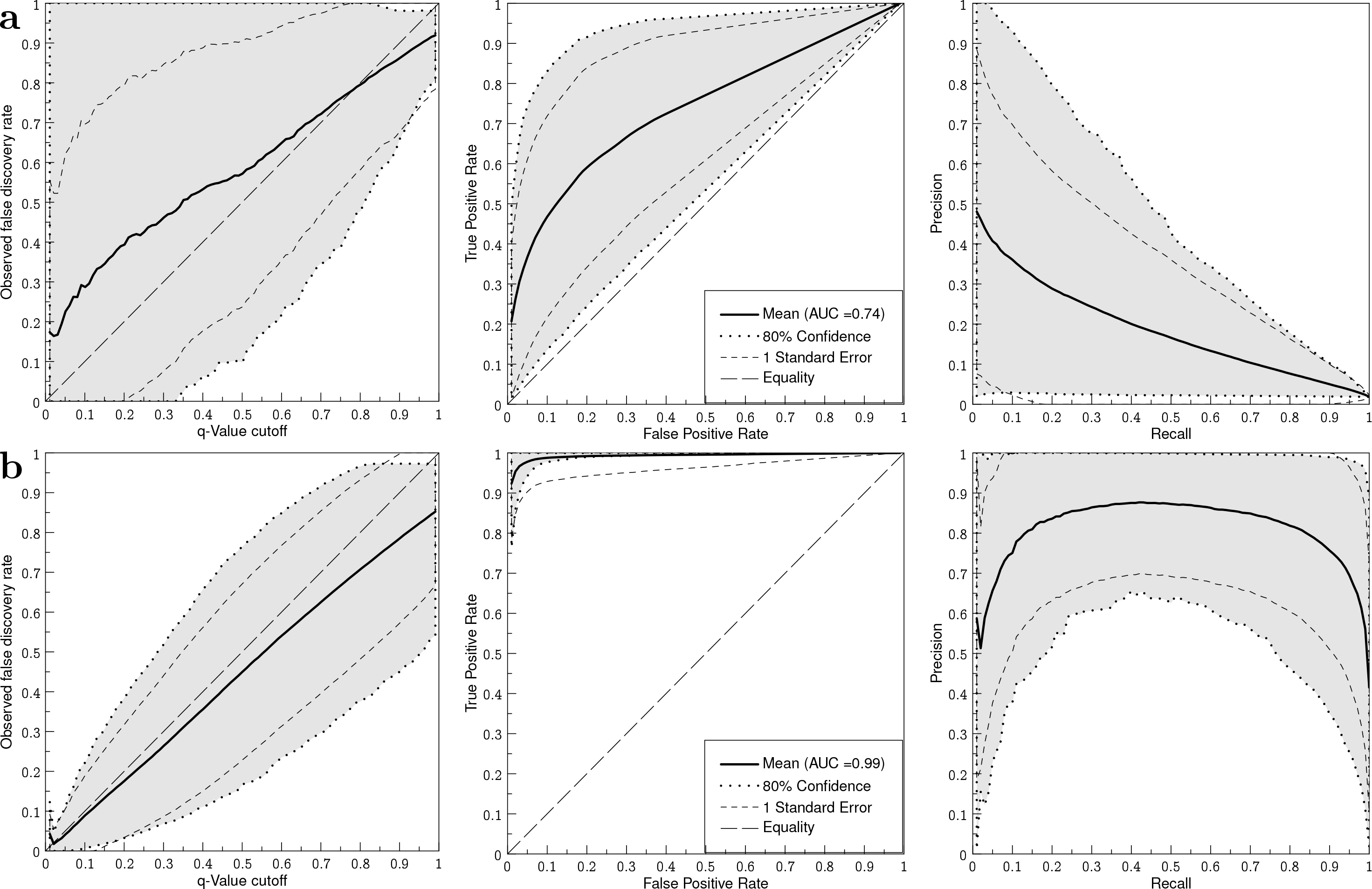

Supplement: S2 Fig — Fisher’s test using a) ∅ 6 cm and b) ∅ 10 cm tumors. Other parameters were at nominal values: 4 × 4 × 4 mm voxels, patient cohorts of 50 patients, a 60% extent of resection, and a ∅ 4 cm effect region with an avoidance probability difference of 0% versus 100%. (TIF) [file pone.0222939.s003.tif]

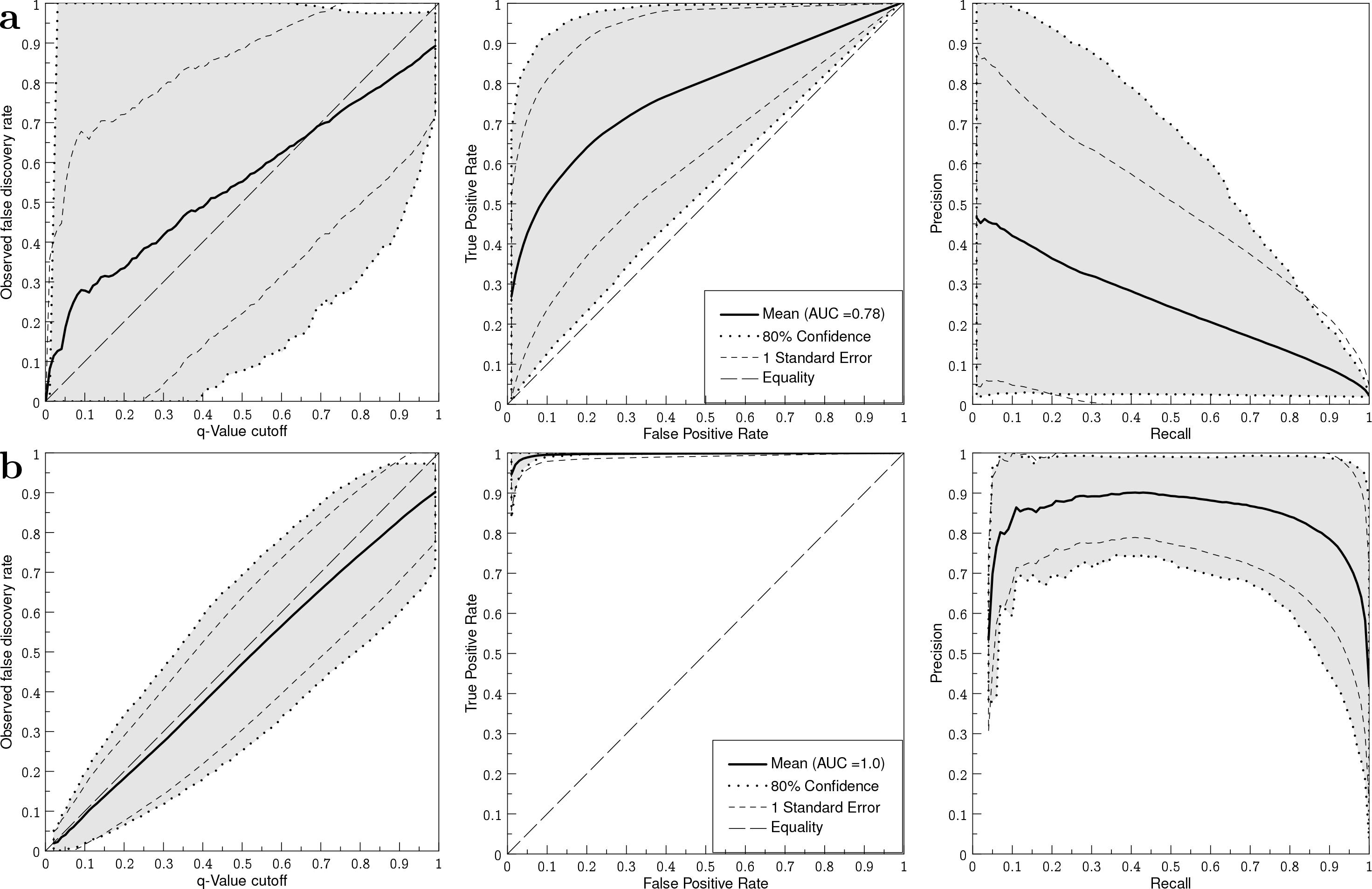

Supplement: S3 Fig — Fisher’s test using a) patient cohorts of 25 patients and b) patient cohorts of 100 patients. Other parameters were at nominal values: 4 × 4 × 4 mm voxels, ∅ 8 cm tumors, a 60% extent of resection, and a ∅ 4 cm effect region with an avoidance probability difference of 0% versus 100%. (TIF) [file pone.0222939.s004.tif]

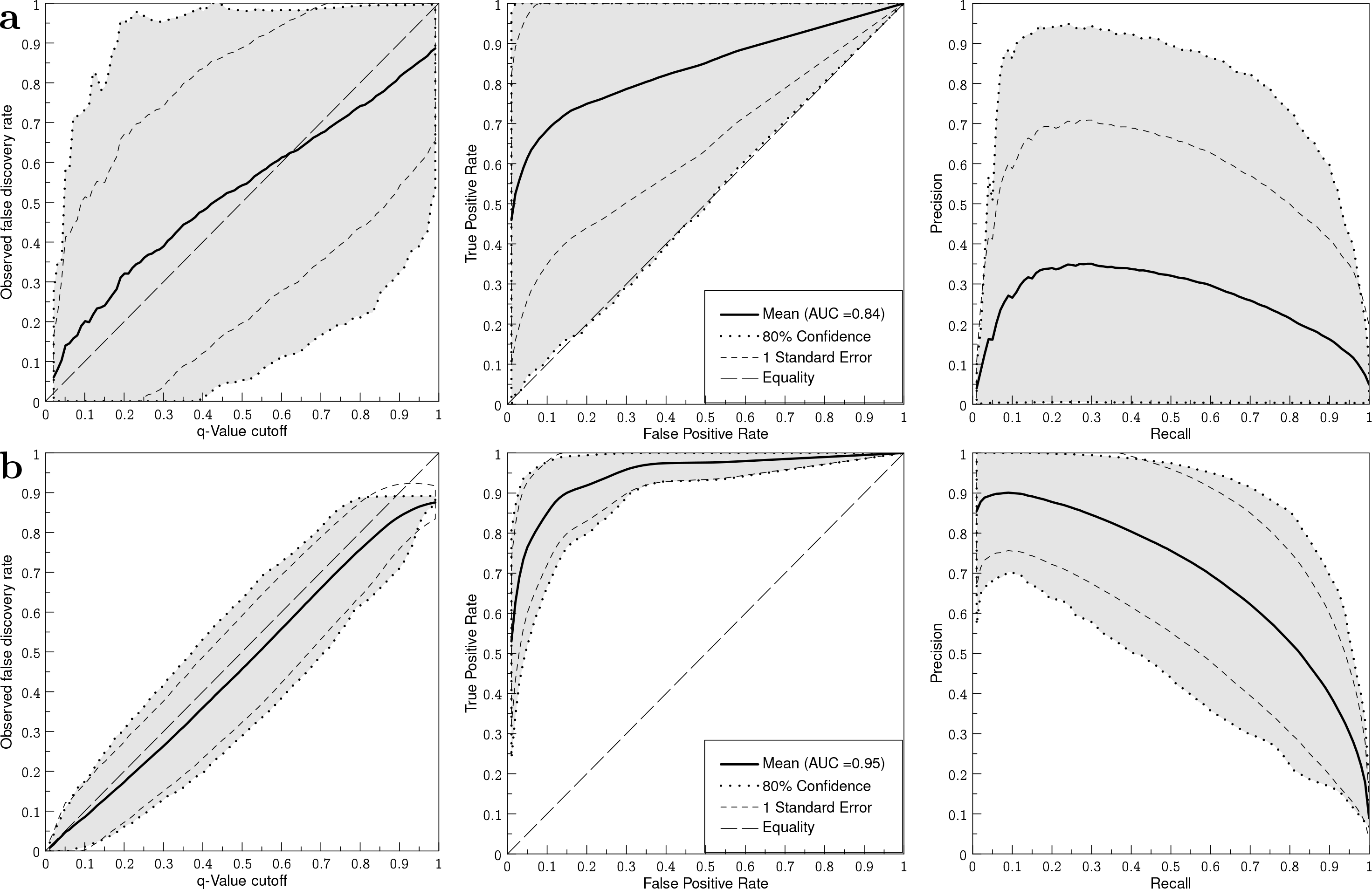

Supplement: S4 Fig — Fisher’s test using a) a ∅ 2 cm and b) a ∅ 6 cm effect region. Other parameters were at nominal values: 4 × 4 × 4 mm voxels, ∅ 8 cm tumors, patient cohorts of 50 patients, a 60% extent of resection, an avoidance probability difference of 0% versus 100% inside the effect region. (TIF) [file pone.0222939.s005.tif]

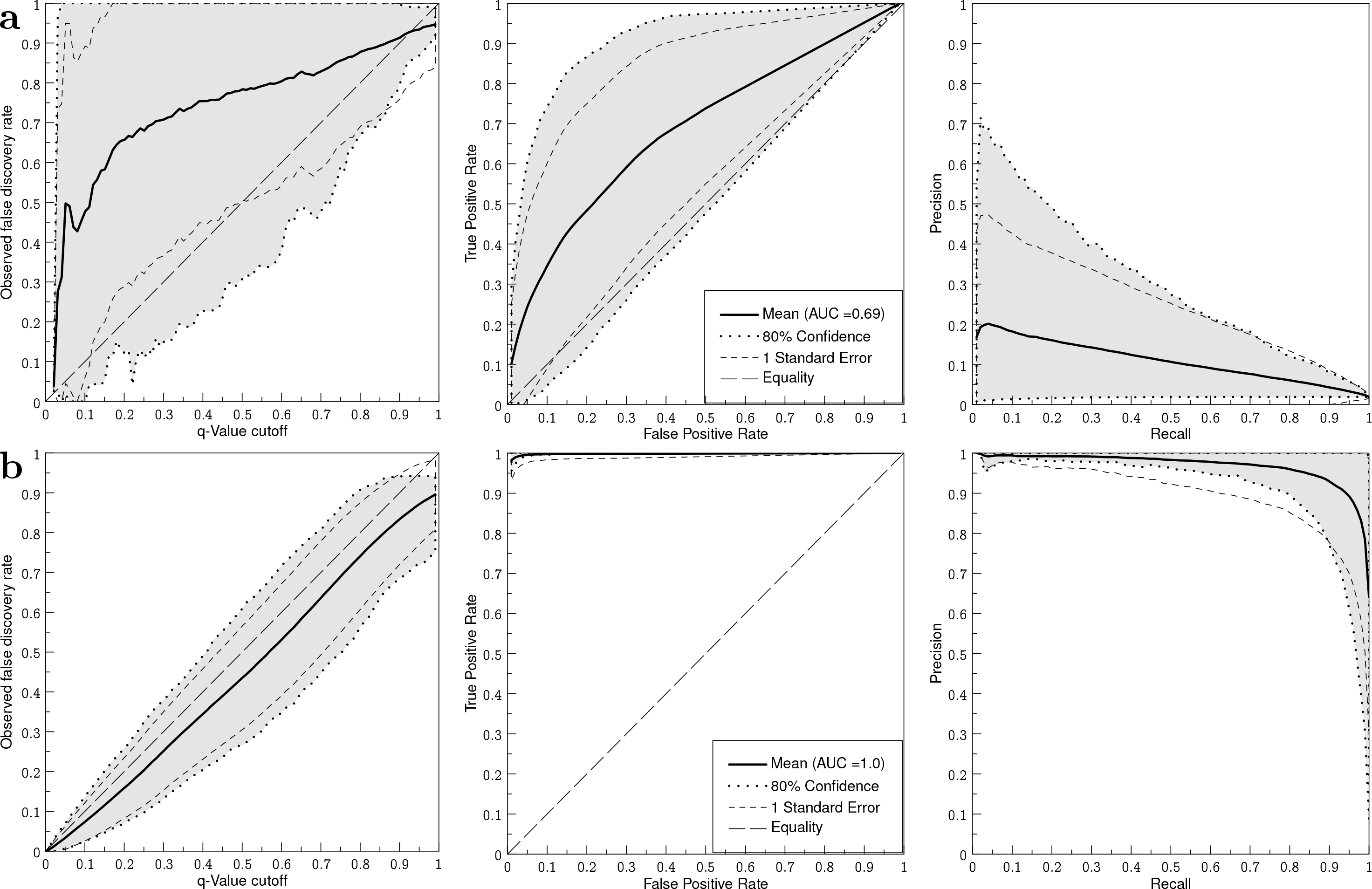

Supplement: S5 Fig — Fisher’s test using a) a 60% and b) a 90% extent of resection. Other parameters were at nominal values: 4 × 4 × 4 mm voxels, ∅ 8 cm tumors, patient cohorts of 50 patients, and a ∅ 4 cm effect region with an avoidance probability difference of 0% versus 100%. (TIF) [file pone.0222939.s006.tif]

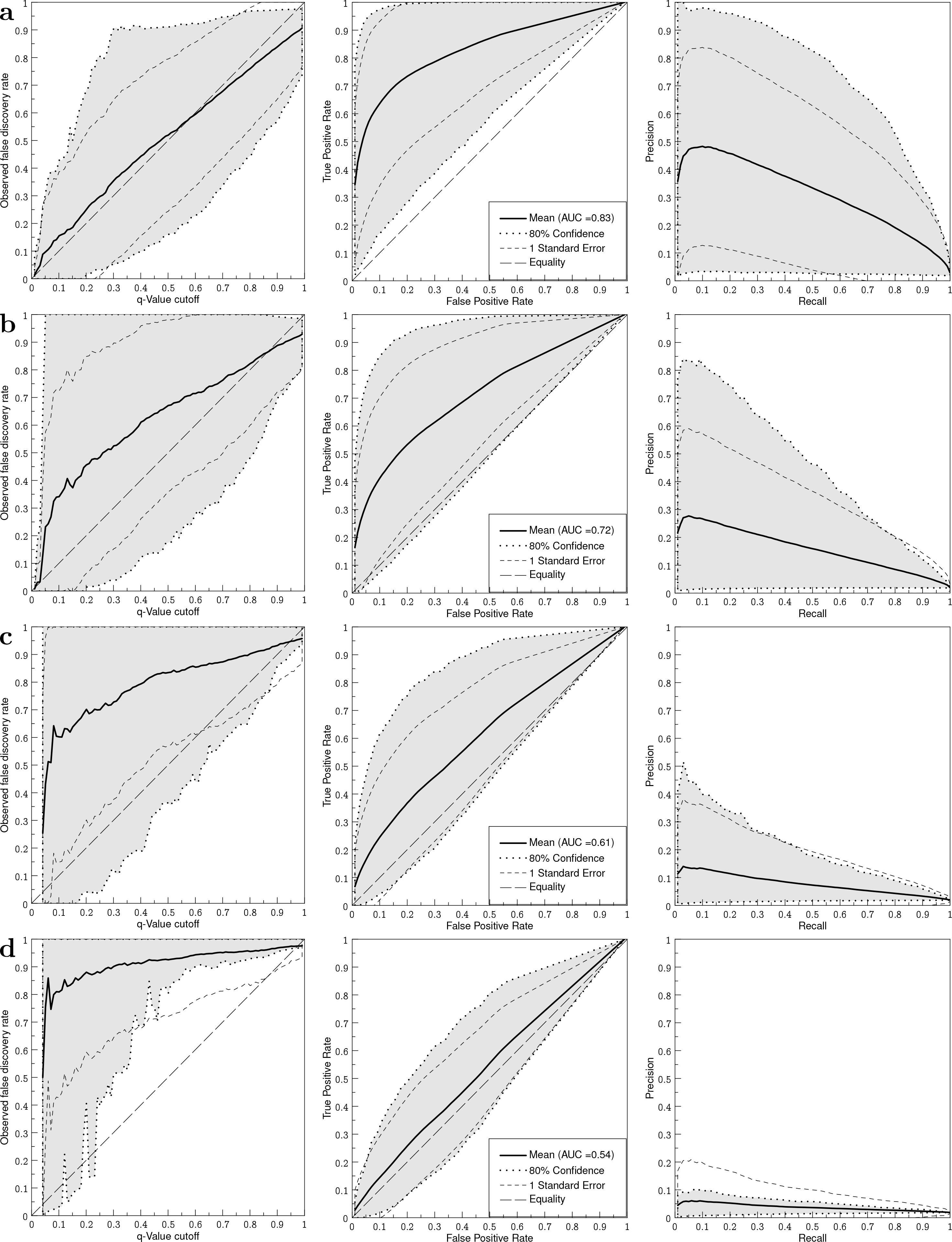

Supplement: S6 Fig — Fisher’s test using avoidance probability differences of a) 10% versus 90%, b) 20% versus 80%, c) 30% versus 70%, and d) 40% versus 60% inside the effect region. Other parameters were at nominal values: 4 × 4 × 4 mm voxels, ∅ 8 cm tumors, patient cohorts of 50 patients, a 60% extent of resection, and a ∅ 4 cm effect region. (TIF) [file pone.0222939.s007.tif]

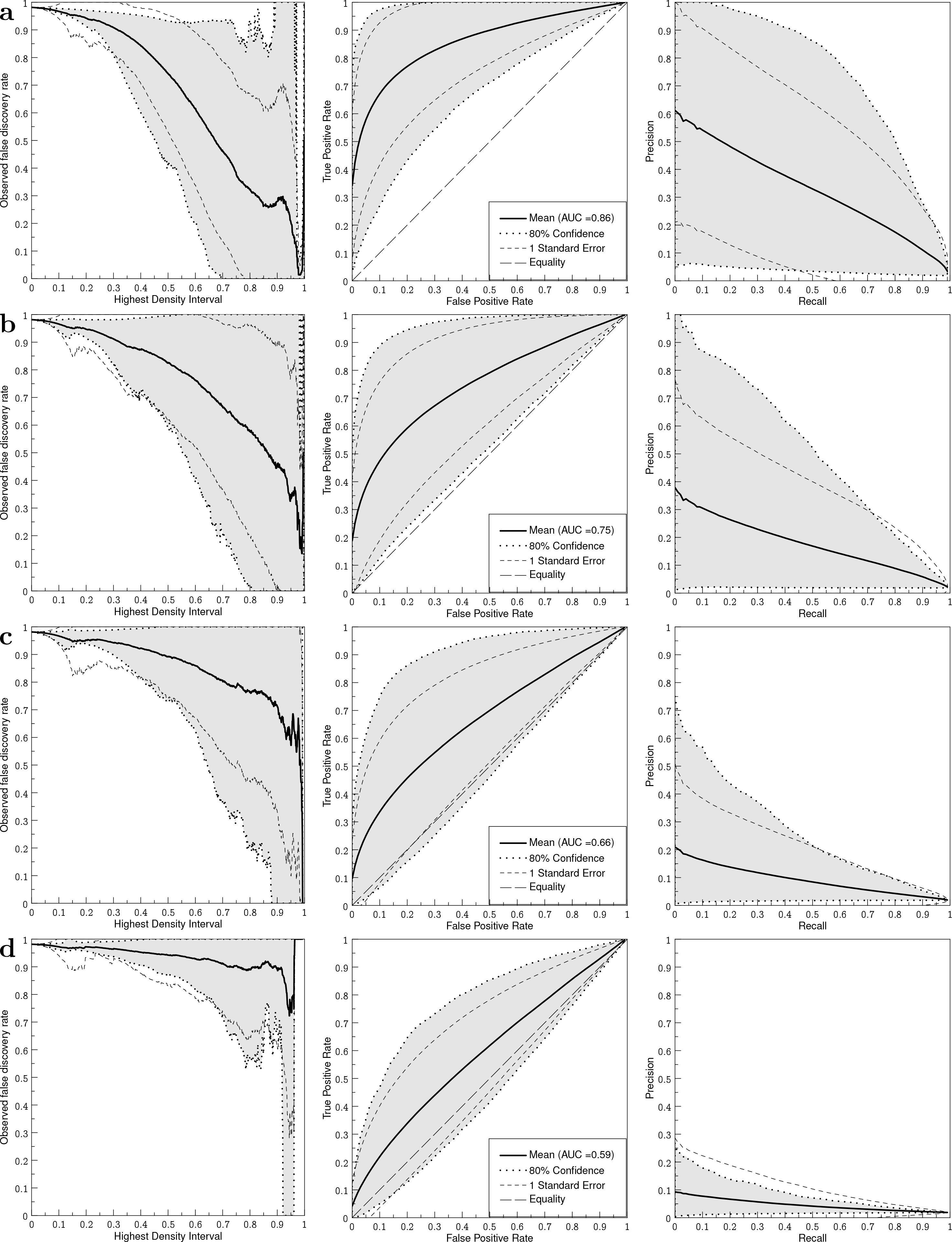

Supplement: S7 Fig — The Bayesian method using avoidance probability differences of a) 10% versus 90%, b) 20% versus 80%, c) 30% versus 70%, and d) 40% versus 60% inside the effect region. Other parameters were at nominal values: 4 × 4 × 4 mm voxels, ∅ 8 cm tumors, patient cohorts of 50 patients, a 60% extent of resection, and a ∅ 4 cm effect region. (TIF) [file pone.0222939.s008.tif]
